# Women’s self-employment, business acumen, and emotional IPV: a longitudinal study in Tanzania

**DOI:** 10.1186/s12905-026-04369-3

**Published:** 2026-03-05

**Authors:** Shruti Shukla, Gerry Mshana, Neema R. Mosha, Janina Isabel Steinert, Joanna Krajewska, Sheila Harvey, Shelley Lees, Saidi Kapiga, Heidi Stöckl

**Affiliations:** 1 TUM School of Social Sciences and Technology, Technical University of Munich, Richard Wagner Strasse 1, Munich, 80333 Germany; 2 Institute for Medical Information Processing, Biometry and Epidemiology, Faculty of Medicine, Ludwig-Maximilians-University, Munich, Germany; 3 Pettenkofer School of Public Health, Munich, Germany; 4 National Institute for Medical Research, Mwanza, Tanzania; 5 Mwanza Intervention Trials Unit, Mwanza, Tanzania; 6 TUM School of Medicine and Health, Technical University of Munich, Munich, Germany; 7 Munich Center for Health Economics and Policy (M-CHEP), Munich, Germany; 8 Department of Global Health and Development, London School of Hygiene and Tropical Medicine, London, UK; 9 Department of Infectious Disease Epidemiology and International Health, London School of Hygiene and Tropical Medicine, London, UK

**Keywords:** Emotional intimate partner violence, Women's empowerment, Tanzania, Women entrepreneurs, Self-employment

## Abstract

**Background:**

Emotional intimate partner violence (IPV) is the most prevalent form of IPV and is associated with severe health consequences for women. Despite its widespread occurrence, emotional IPV remains under-researched, particularly in relation to women’s economic empowerment. While prior studies have examined the role of cash transfers and microfinance, less is known about the possible protective effects of self-employment and business acumen on emotional IPV.

**Methods:**

Using data from the MAISHA longitudinal study, we examined how indicators of business acumen - including business ownership, leadership, working hours, and reinvestment of earnings - relate to women’s experiences of emotional IPV, measured via a composite index in Mwanza, Tanzania. In a mixed‑effects logistic regression model, we include business acumen and emotional IPV measures at each of four waves (*N* = 1,004 at Wave 1 & *N* = 836 (83%) at Wave 4), adjusting for sociodemographic covariates.

**Results:**

Across all waves, approximately 80% of economically active women were self-employed. Emotional IPV was commonly reported, with an increasing trend over time, rising from 44.8% at Wave 1 to 52.7% at Wave 4. The intra-class correlation (ICC) of 35% indicated stability in IPV experiences between women, while 65% reflects within-woman variability over time. In adjusted models, women who held leadership roles within their businesses (AOR = 0.69; 95% CI: 0.49–0.97) and those who reinvested earnings (AOR = 0.66; 95% CI: 0.53–0.82) had significantly lower odds of experiencing emotional IPV.

**Conclusion:**

Findings suggest that among self-employed women, business leadership and financial reinvestment may serve as protective factors against emotional IPV. Enhancing women’s business capacity may strengthen their agency and reduce IPV risk in contexts with high female labour force participation.

**Supplementary Information:**

The online version contains supplementary material available at 10.1186/s12905-026-04369-3.

## Introduction

Emotional intimate partner violence (IPV) is the most prevalent form of IPV, yet it remains understudied compared to physical and sexual IPV. According to global estimates by the World Health Organisation (WHO), approximately 30% of women worldwide have experienced physical and/or sexual IPV in their lifetime, compared to nearly 50% who report experiencing emotional IPV [1–3]. Prevalence rates of emotional IPV among women range from 9% to 70%, depending on cultural and contextual factors [4].

A multitude of terms are used interchangeably for emotional IPV, including psychological violence or emotional abuse. Accordingly, its definition has also evolved over time. Earlier conceptualizations included a broad range of behaviours such as verbal abuse, threats of violence, humiliation, jealousy, flirting with other sexual partners, damage to the partner’s self-image, withholding of emotional support, monitoring partner movements, and restricting personal territory or freedom as forms of emotional IPV [5, 6]. However, more recent research distinguishes emotional IPV from male controlling behaviours. A cross-cultural study using data from the World Health Organisation (WHO) Multi-Country Study on Domestic Violence and Women’s Health across 10 countries confirmed that psychological abuse and male controlling behaviours are distinct constructs and should not be combined in measurement or analysis [7]. Consequently, this paper adopts the WHO’s widely used definition of emotional IPV, which includes “insults, belittling, constant humiliation, intimidation (e.g., destroying things), threats of harm, and threats to take away children” [8].

Emotional IPV often co-occurs with other forms of violence, including physical and sexual IPV, stalking, and controlling behaviours. A recent meta-analysis found significant associations between emotional IPV perpetration and victimization with these forms of violence [9]. Previous studies have also demonstrated that emotional violence is a precursor of many forms of IPV [10–14]. Additionally, emotional IPV has been linked to poor mental and physical health outcomes for women. Both systematic reviews and individual studies have shown that women who experience emotional IPV often report adverse health outcomes like depression, suicidal ideation, post-traumatic stress, anxiety, physical pain, and poor physical health [15–18]. Specifically, in Tanzania, studies have shown high rates of emotional IPV reported by both survivors and perpetrators, with significant associations with poor mental health for both partners [19–21].

Given the high prevalence and severe consequences of emotional IPV, it is crucial to explore protective factors that may mitigate its impact. One such factor is women’s economic empowerment, particularly through access to economic resources. Several experimental studies in Africa have found that improved access to economic resources can reduce women’s IPV experience, including emotional IPV [22–26]. Explaining this relationship, bargaining theory suggests that a woman’s higher income can reduce violence by alleviating financial stress and increasing her decision-making power within the household [27–30]. However, conversely, changing established gender norms relating to financial power may lead to “male backlash”, as male partners perceive a threat to their authority and control, potentially leading to increased conflict and IPV perpetration [31, 32].

Further, most existing research focuses on economic interventions such as cash transfers or microfinance, leaving the specific role of self-employment in IPV prevention underexplored. Self-employment can offer women greater autonomy, control over working hours, and direct decision-making power over financial resources, which may influence IPV risk differently. It can also help women to manage their household and financial responsibilities more independently, thus increasing empowerment. In addition to financial independence, being self-employed allows women to build hard and soft skills, such as assertiveness, leadership, motivation, self-confidence, and resilience, which are critical for running a business and navigating household power dynamics [33]. Women in Tanzania mainly engage in self-employment activities like retailing, catering, food vending, and hair salons, with capital sources ranging from their own savings, semi-formal financial sources like village community banks, microfinance institutions, and savings and credit cooperatives, as well as informal channels like loans from family and friends [34].

Tanzania presents a suitable context for examining the association between self-employment and IPV, given that women entrepreneurs are vital in driving economic growth and societal development in the country [34]. A qualitative study in Tanzania further highlighted that women’s independent income can stabilise households and improve partner relationships without increasing IPV exposure [35]. However, other studies conducted in different African countries, where half of all entrepreneurs are women [33, 36], have not found a clear association between women’s entrepreneurship and IPV. They suggest that contextual factors such as spousal awareness of women’s economic activities, household decision-making dynamics, and cultural norms play a crucial role in shaping outcomes [37–39].

Given the limited research on this topic, we examine how women’s self-employment and related business acumen indicators are associated with their emotional IPV experiences in Mwanza, Tanzania, using longitudinal data over five years. We define business acumen as women’s degree of active engagement and decision-making authority within self-employment, rather than business performance or success. Accordingly, our focus is on dimensions of economic agency, such as leadership in business, sustained time investment, and control over the use of business earnings. We hypothesise that women’s business acumen is negatively associated with their experience of emotional IPV in the past 12 months. Empowerment and agency gained through self-employment and business activities may enhance their bargaining power and self-esteem, reducing vulnerability to IPV. Notably, Tanzania’s high female labour force participation, 80.2% for women compared to 87.4% for men, suggests that women’s economic engagement may be perceived as less threatening to traditional gender roles than in some Asian contexts [40]. Thus, indicating that there is a level of normative acceptance for women’s entrepreneurship in Tanzania. This minimizes confounding due to stigma and allows us to test whether specific indicators of women’s economic participation are associated with reduced emotional IPV when women’s work itself is common. Furthermore, 76% of employed women and 68.3% of employed men work in the informal sector, where self-employment is most common. In contrast, the formal sector is more male-dominated, with 71% of jobs held by men and only 35% by women [40, 41]. These figures underscore the relatively small gender disparities in the informal sector, reinforcing the relevance of examining self-employment in this context.

## Methods

### Study design

This study was conducted in Mwanza City, located in Northwest Tanzania. The MAISHA longitudinal study utilised data from the women in the control groups of two cluster randomised controlled trials of a social empowerment intervention. Full details of the intervention have been reported elsewhere [23, 42, 43]. The MAISHA study then followed up with the same women twice after the intervention. Baseline interviews collected between September 2014 and July 2015 comprised Wave 1 of the study. Outcomes were collected two years post-intervention between May 2017 and January 2018, making Wave 2. After Wave 2, the control group women were asked if they would participate in a follow-up study (the MAISHA longitudinal study). Those who agreed were interviewed at 41 months (between July 2018 and March 2019) and 53 months (between October 2019 and March 2020) from baseline (Wave 3 and Wave 4). The retention rate was approximately 80% in all the waves. The main reasons for loss to follow-up were death, migration, or withdrawal from the study.

Data were collected through face-to-face interviews by trained female interviewers fluent in Swahili at a private location and lasted approximately 1.5–2 h. The survey instrument was first developed in English and then translated into Swahili. It covered various themes, including household details, employment, income, health, and IPV experiences (see supplementary file 1). Data was uploaded daily to ensure data integrity. This analysis includes women in the control group in all four waves who indicated being in a relationship in the last 12 months. The assessment of past-year emotional IPV exposure was limited to this subgroup. Women were asked: “Are you married or presently cohabiting with a man?“. If the response was negative, they were asked, “Have you been in a relationship with a man within the past 12 months?” and included in the sample if they affirmed.

The study adhered to ethical guidelines recommended by the World Health Organisation (WHO) for researching violence against women [44]. Informed consent procedures were followed at all four waves of data collection. All participants received information about the study’s purpose and provided either written or verbal consent to participate. The longitudinal study received ethical approval from the Tanzanian National Health Research Ethics Committee (Ref: NIMR/HQ/R.8a/Vol.IX/2475), the Ludwig Maximilian University ethics committee (21–0507) and the London School of Hygiene and Tropical Medicine (Ref: 11,918–4).

### Study Setting

The study was conducted in Mwanza City, Tanzania’s second largest urban centre, on the southern shores of Lake Victoria [43]. The population is predominantly low-income and ethnically diverse, with livelihoods that are largely informal and economically insecure [45]. Men commonly work in fishing, construction, casual labour, and motorcycle taxi services (bodaboda), while women’s economic activities centre on petty trading, tailoring, and small-scale agriculture, often supported through microfinance groups [46].

Intimate relationships are shaped by patriarchal norms, with marriage commonly formalised through bride price and extramarital partnerships remaining prevalent [47]. Gender norms position men as primary providers and women as responsible for domestic labour and childcare, although women’s increasing economic participation has generated household tensions [48]. IPV is widespread and frequently linked to romantic jealousy, perceived challenges to male authority, and deviations from expected gender roles, with social norms often encouraging women to tolerate violence to preserve family cohesion [47].

### Measures

#### Dependent variable: Emotional IPV

The emotional IPV outcome measures were based on standardised measures adapted from the WHO Multi-Country Study Instrument [8]. We assessed women’s experiences of male partner-perpetrated emotional IPV in the past 12 months.

We utilise a dummy binary variable of any current emotional IPV denoting 1 if any of the below four questions were answered as ‘Yes’, otherwise it was coded as 0. We define this as the composite emotional IPV outcome.

We consider individual items of emotional IPV with binary responses, including: Has your current or last husband/partner done the following to you in the past 12 months?

1. Insult – Insulted you or made you feel bad about yourself?
2. Humiliate – Belittled or humiliated you in front of other people?
3. Scare – Done things to scare or intimidate you on purpose (e.g. by the way he looked at you, by yelling and smashing things)?
4. Threat – Verbally threatened to hurt you or someone you care about?

Throughout the study, respondents who reported experiencing IPV were provided with information and referrals to support services within their communities, ensuring ethical considerations regarding participant well-being.

#### Independent variables: Self-employment and Business acumen

In this study, business acumen is conceptualised as women’s economic agency in self-employment, reflecting the extent to which women exercise control, take on responsibility, and engage in strategic decision-making in their business activities. The selected indicators measure women’s leadership in business operations, continuity of engagement, time investment, and discretion over the allocation of business earnings. These dimensions capture meaningful involvement in economic activity and proxy women’s capacity to exercise autonomy and bargaining power within informal self-employment contexts.

To examine the subset of self-employed women within the sample, participants were first asked whether they had personally earned money in the past 12 months. Those who responded affirmatively were then asked whether they were self-employed or worked for someone else. The analysis was conducted on women who identified as self-employed.

Four key binary indicators were used to assess the business acumen of self-employed women:

1. Business Ownership – Participants reported whether they had been operating their business for more than 12 months.
2. Leadership – Women indicated whether they were primarily responsible for managing their business.
3. Working Hours – Respondents specified the number of hours they typically worked per day, categorized as: less than 8 h or more than 8 h.
4. Investment – Participants reported if they reinvested their earnings in their business over the past month.

#### Control variables

Several socio-demographic variables were examined as covariates and possible confounding factors in examining the link between women’s business acumen and emotional IPV. These variables included women’s age, partner’s age, partner’s employment status in the last 12 months, cohabitation status, and socioeconomic status (SES). The age variables were not collected at Wave 2 and 3 and were calculated based on Wave 1.

### Analysis

All analyses were conducted using R statistical programming software [49]. Descriptive statistics were computed to summarise the prevalence of individual emotional IPV items and the binary composite emotional IPV variable at each survey wave. The prevalence of business acumen indicators was assessed at each wave, and differences in proportions across waves were examined using McNemar’s chi-square test for binary variables and Wilcoxon test for categorical variables. These comparisons are presented for descriptive and contextual purposes to characterise temporal variation. Bivariate mixed-effects logistic regression models were used to examine unadjusted associations between each business acumen indicator and the binary composite emotional IPV outcome. All bivariate models included participant ID as a random effect to account for individual-level variation across time. Models were also estimated using individual emotional IPV items as outcomes. However, due to sparse endorsement of some items across waves, these mixed-effects logistic models exhibited instability and failed to converge. Therefore, we report all statistical analyses with the composite measure of emotional IPV only.

Final multivariable analyses employed mixed effects logistic regression models with a logit link, estimated within the sub-sample of women who reported self-employment in the past 12 months (*N* = 839). Business acumen indicators were included simultaneously as fixed effects in the adjusted models. A participant-level random intercept was specified to account for within-person correlation across repeated observations using a unique participant ID. Fixed effects also included survey wave indicators, which were incorporated to control for wave-specific contextual effects and changes in emotional IPV prevalence rather than to model individual temporal trajectories. All four waves were pooled in a longitudinal format to account for repeated measures. The mixed-effects framework accommodates unbalanced longitudinal data; women with only one observation were also retained in the analysis. Participants with data across two or more waves contributed to the estimation of within-person correlations, while those with a single observation contributed only to between-person estimation and were not excluded from the model. Both unadjusted and adjusted models were estimated, with adjusted models controlling for pre-specified socio-demographic covariates. The full model specification, including R syntax for all mixed-effects models, is publicly available via the Open Science Framework (OSF).

## Results

The final analytical sample comprised 1,004 ever-partnered women at Wave 1, 892 at Wave 2, 867 at Wave 3, and 836 at Wave 4. Table 1 presents the socio-demographic characteristics of the sample. The age of participating women ranged from 18 to 70 years (mean = 38; SD = 9.2), while their partners ranged from 19 to 96 years of age (mean = 45; SD = 15.7).

**Table 1:** Socio-demographic characteristics

Characteristics	Overall, *N* = 3,599^1^	Wave			
		1, *N* = 1,004^1^	2, *N* = 892^1^	3, *N* = 867^1^	4, *N* = 836^1^
Women’s Mean Age (SD)	38 (9.2)	35 (8.9)	39 (8.8)	41 (8.9)	39 (9.0)
Partner’s Mean Age (SD)	45 (15.7)	42 (17.3)	45 (14.5)	48 (14.0)	47 (16.0)
Married/Co-habiting					
Yes	3,039 (84.4%)	859 (86.6%)	755 (84.6%)	736 (84.9%)	689 (82.4%)
No	560 (15.6%)	145 (14.4%)	137 (15.4%)	131 (15.1%)	147 (17.6%)
Social Economic Status Quantile Score					
First quantile (lowest)	690 (20%)	199 (20%)	170 (20%)	163 (20%)	158 (20%)
Second quantile	722 (21%)	202 (20%)	179 (21%)	174 (21%)	167 (21%)
Third quantile	696 (20%)	201 (20%)	173 (20%)	165 (20%)	157 (19%)
Fourth quantile	692 (20%)	200 (20%)	169 (20%)	160 (19%)	163 (20%)
Fifth quantile	709 (20%)	202 (20%)	175 (20%)	168 (20%)	164 (20%)

^1^Count (n) or Frequency (%), Overall N represents the pooled sample size across four waves

Across the four waves, 873 women at Wave 1, 779 at Wave 2, 779 at Wave 3, and 763 at Wave 4 reported engagement in some form of economic activity in the previous 12 months. Of all women, 82.0%, 79.0%, 79.2%, and 80.8%, respectively, identified as self-employed at each wave (Table 2). Within the subsample (*n* = 823) of self-employed women at Wave 1, 75% reported owning a business, 91% were primarily responsible for the business, 58% worked more than eight hours per day, and 39% reinvested part of their earnings into the business (Appendix Table 1). The rates varied across the four time points, with significant differences between Wave 1 and Wave 3 or 4 (Table 2). Overall, women’s economic activity increased across the four waves, while self-employment rates remained relatively stable.

**Table 2:** Prevalence of the various business acumen variables in the whole sample

Independent variables	Wave			
	1, *N* = 1,004^*1*^	2, *N* = 892^*1*^	3, *N* = 867^*1*^	4, *N* = 836^*1*^
Economic activity in the past 12 months	873 (87%)	779 (87%)	**779 (90%)** ^*^	**763 (91%)** ^*^
Employment type				
Self-employed	823 (82.0%)	**706 (79.0%)** ^*^	**687 (79.2%)** ^*^	**676 (80.8%)** ^*^
With an organisation	34 (3.4%)	**64 (7.1%)** ^*^	**79 (9.1%)** ^*^	**57 (6.8%)** ^*^
Both	16 (1.5%)	**9 (1.0%)** ^*^	**13 (1.5%)** ^*^	**20 (2.4%)** ^*^
Not employed	0 (0%)	**0 (0%)** ^*^	**0 (0%)** ^*^	**10 (1.2%)** ^*^
Ownership of business	632 (63.0%)	555 (62.2%)	559 (64.4%)	**565 (67.6%)** ^*^
Leadership of business	761 (75.8%)	**629 (70.5%)** ^*^	641 (73.9%)	**579 (69.3%)** ^*^
Work hours				
0–4 h	170 (17.0%)	**112 (12.5%)** ^*^	**79 (9.1%)** ^*^	132 (15.7%)
5–8 h	285 (28.4%)	**250 (28.0%)** ^*^	**274 (31.6%)** ^*^	242 (28.9%)
More than 9 h	364 (36.2%)	**349 (39.1%)** ^*^	**347 (40.0%)** ^*^	326 (39.0%)
More than 8 h (binary)	458 (45.6%)	**432 (48.4%)**	**425 (49.0%)**	390 (46.7%)
Investment for business				
None of it	374 (37.2%)	371 (41.6%)	364 (42%)	**338 (40.4%)** ^*^
Some of if	219 (21.8%)	180 (20.2%)	206 (23.8%)	**234 (28%)** ^*^
Half of it	69 (6.8%)	54 (6%)	82 (9.4%)	**57 (6.8%)** ^*^
Most of it	29 (2.9%)	29 (3.2%)	38 (4.3%)	**22 (2.6%)** ^*^
All of it	10 (0.9%)	2 (0.2%)	10 (0.1%)	**3 (0.3%)** ^*^
Any investment (binary)	327 (32.6%)	265 (29.7%)	336 (38.8%)	**316 (37.8%)**

^*1*^Count (n) or Frequency (%)

*Boldface indicates McNemar’s Χ^2^ test or Wilcoxon test at p-value < 0.05, indicating a significant change in prevalence between waves. Wilcoxon test was used for categorical variables and McNemar for binary variables

Regarding emotional IPV, 44.8%, 47.4%, 47.8%, and 52.7% of self-employed women across the four waves (over 5 years) reported experiencing at least one form of emotional IPV, defined as composite emotional IPV (Table 3). Detailed information on each emotional IPV item and composite measures across the sub-sample and the full sample is provided in Table 3 and Appendix Table 2, respectively. Reports of composite emotional IPV and partner insults showed an increasing trend over time. Significant differences were observed in the prevalence of composite emotional IPV across waves. However, only the insult item from the emotional IPV scale showed a statistically significant difference between Wave 1 and Wave 4.

**Table 3:** Prevalence of emotional IPV for the sub-sample of self-employed women

Outcome variable	Wave			
	1, *N* = 839^*1*^	2, *N* = 715^*1*^	3, *N* = 700^*1*^	4, *N* = 696^*1*^
Insult	341 (40.6%)	305 (42.7%)	**313 (44.7%)** ^*^	**351 (50.4%)** ^*^
Humiliate	130 (15.5%)	127 (17.8%)	**102 (14.6%)** ^*^	92 (13.2%)
Scare	157 (18.7%)	141 (19.7%)	**105 (15%)** ^*^	101 (14.5%)
Threat	170 (20.2%)	147 (20.5%)	**119 (17%)** ^*^	111 (15.9%)
Composite emotional IPV	376 (44.8%)	339 (47.4%)	335 (47.8%)	**367 (52.7%)** ^*^

^*1*^Count (n) or Frequency (%)

*Boldface indicates McNemar’s Χ^2^ test at p-value < 0.05, indicating a significant change in prevalence between waves

Bivariate regression analyses indicated a significant negative association between composite emotional IPV and the four key independent variables: business ownership, leadership, working hours, and reinvestment of earnings (Appendix Table 3). Unadjusted multivariable analyses were conducted to identify significant relationships to be included in the adjusted model (Appendix Table 4). All variables except for business ownership were significantly associated with composite emotional IPV.

The final adjusted multivariable mixed-effects model, controlling for women’s age, partner’s age, socioeconomic status, cohabitation status, and Wave, is presented in Table 4. Since the partner’s employment status in the past 12 months had many missing values and was not significantly associated with composite emotional IPV in the bivariate regression model, we did not include it in the final analysis.

**Table 4:** Adjusted multivariable model for composite emotional IPV and finance acumen variables

Composite Emotional IPV			
Business Acumen Indicators	AOR^1^	95% CI^1^	*p*-value
Leadership	0.69	0.49, 0.97	**0.032**
More than eight hours of work	0.84	0.67, 1.06	0.14
Any investment	0.66	0.53, 0.82	**< 0.001**
Covariates			
SES	0.93	0.85, 1.02	0.14
Cohabitation status	0.50	0.35, 0.71	< 0.001
Woman’s Age	0.69	0.57, 0.84	< 0.001
Partner’s Age	0.95	0.77, 1.18	0.7
Wave	1.19	1.09, 1.31	< 0.001
ICC	0.35		
AIC	3112.19		
Deviance	2166.25		

The goodness of fit is reported as AIC (Akaike information criterion) and Deviance

^1^*AOR*  Odds Ratio, *CI*  Confidence Interval. Boldface indicates *p*-value <0.05

Subsample of self-employed women. Dummy variable for Wave is included as a fixed effect

Results indicated that self-employed women in leadership roles (AOR = 0.69; 95% CI: 0.49–0.97, *p* = 0.03) and those who reinvested earnings into their business (AOR = 0.66; 95% CI: 0.53–0.82, *p* < 0.001) had significantly lower odds of experiencing composite emotional IPV. The intra-class correlation coefficient (ICC) was 0.35 for the mixed-effects model, indicating that approximately 35% of the variance in the latent propensity to experience emotional IPV was attributable to between-individual differences, with the remaining 65% variance reflecting within-individual variation over time.

## Discussion

This longitudinal study examined the relationship between women’s business acumen and emotional intimate partner violence in Mwanza, Tanzania, over five years. Notably, while overall economic activity and self-employment rates among women increased across the four waves, the prevalence of emotional IPV rose as well. Our findings demonstrate that specific dimensions of business acumen, namely leadership within the business and reinvestment of earnings, are significantly associated with lower odds of experiencing emotional IPV. These associations remained robust even after controlling for socio-demographic factors, underscoring the importance of differentiating between types of economic engagement and their distinct implications for emotional IPV risk.

Our findings contribute to a growing body of literature from low- and middle-income countries on the links between women’s economic empowerment and IPV. While some studies suggest that women’s increased income may provoke backlash in patriarchal settings, especially when men perceive a threat to traditional gender roles [50–53], others find that economic interventions can reduce IPV, particularly when combined with gender-transformative components or when the partners have relatively higher incomes [22, 54, 55]. Though much of this evidence is focused on sexual and/or physical IPV.

The mixed evidence likely reflects differences in the nature and quality of women’s economic engagement as well as the context and social norms around female employment. Our findings reinforce the notion that economic engagement alone is not inherently protective, rather, empowerment that promotes meaningful control and decision making for women is key. Women who take on leadership roles or reinvest earnings may develop greater self-efficacy and bargaining power within relationships, reducing their susceptibility to emotional abuse. A qualitative study on women’s paid work in the informal sector and IPV in Tanzania showed that women aligned their own self-interest with that of their family, thereby exercising agency and starting their own business [56]. Here, it is important to consider the source of capital, like formal loans from microfinance institutions or from family for self-employment activities. It is of importance that the funds are controlled by the women themselves, as this can further enhance their agency. Microfinance loans that require the guarantee of male partners, for example, can be problematic, as they may undermine women’s financial autonomy. Further, another economic empowerment intervention study in Burkina Faso showed an increase in women’s financial autonomy and a subsequent reduction in emotional spousal violence [24].

These findings align with empowerment and bargaining theories, which propose that women’s access to and control over economic resources can shift intra-household dynamics in their favour [27, 30, 31]. Recent studies on business skills training interventions targeted at women show that they can increase women’s autonomy and decision-making power within the family. These interventions have also been shown to increase women’s investments in their businesses, leading to more assets, income and savings in the family, and improve the quality of the relationship between couples [38, 57, 58]. Furthermore, evidence from sub-Saharan Africa, including interventions like MAISHA in Tanzania and IMAGE in South Africa [25, 43], underscores the importance of pairing economic support with increased knowledge about gender rights, confidence and skills-building, or partner involvement to mitigate potential backlash and strengthen IPV prevention outcomes [59].

Running a profitable business or earning an income may also lead to increased economic security, thereby reducing income-related stress and associated triggers of IPV, such as conflict over limited resources. For instance, an employment intervention in Ethiopia that provided women with job offers led to an increase in their income and a decrease in emotional IPV six months post-intervention [60]. In our study, women who reinvested business earnings likely enhanced their household’s economic resilience, potentially reducing financial strain and the emotional tensions that can contribute to IPV. Evidence from the MAISHA trial indicates that higher income was associated with reduced household hardship, fewer arguments over the partner’s inability to provide for the family, improved relationship dynamics, and increased likelihood of relationship dissolution [61]. Potentially due to these pathways, in this sample, increased income was associated with reduced risk of past-year physical and sexual IPV. Lastly, the lack of a significant association between business ownership and emotional IPV suggests that mere ownership without active control may be insufficient for fostering protective effects. Similarly, working long hours was not significantly associated with reduced IPV in adjusted models, indicating that time investment alone may not translate into greater agency.

The findings of this study may be better understood when situated within the gendered labour dynamics of urban Tanzania. Despite disparities in formal employment, women in Tanzania have a high labour force participation rate and are significantly engaged in the informal economy in comparison to women in similar settings, for example, in South Asia, with informal employment rates for women even slightly exceeding those of men [41]. Women commonly work as contributing family workers, particularly in urban areas such as Mwanza, where running small businesses is relatively common [40]. Relatedly, a multi-country study on female employment and spousal abuse found that women entering the labour market in regions where it is common for women to work are less prone to violence [62]. While a larger share of men are employers in companies or household market enterprises in Tanzania, women’s widespread engagement in informal self-employment suggests that such roles are socially normative and may not directly challenge traditional gender roles. A seminal paper by Atkinson et al. [63] sheds light on the interaction between gender norms and IPV. They show that low-income husbands were not more or less likely to abuse their wives than high-income husbands. However, the husband’s relative earnings were negatively and significantly related to the likelihood of abuse, primarily when they had traditional attitudes toward women’s employment. In this study’s socio-cultural context, women’s economic roles may afford them more bargaining power and resilience against emotional abuse.

There are some limitations to consider in this study. Missing data for some individual items on emotional IPV prevented item-level analysis, affecting the nuances of the relationships studied here. The reliance on self-reported IPV and business acumen data introduces the possibility of response bias. Additionally, the generalizability of the findings is limited to Mwanza or similar urban settings, and the study cannot establish causal relationships due to its observational design. The lack of data on income and husband’s gender attitudes restricted our ability to test economic theories, though the husband’s employment status was not significantly associated with emotional IPV. However, this study has several strengths, including its longitudinal design, which allows for the examination of the relationship between women’s business acumen and emotional IPV over time. The use of unique economic empowerment indicators, such as business ownership, leadership within business, working hours and reinvestment of earnings, provides a nuanced understanding of empowerment beyond income generation. By investigating the association between these proxies of economic empowerment and emotional IPV, this study fills a gap in the literature.

In conclusion, our findings highlight the importance of supporting women’s meaningful economic engagement, particularly through self-employment, business leadership and reinvestment, for reducing emotional IPV. Economic interventions, for example, cash transfer programs targeted at women, could provide a foundation for investing or entrepreneurship support as add-on modules to strengthen women’s leadership potential. Governments should also prioritise reducing gender gaps in formal employment by promoting women’s access to secure, well-paying jobs in the formal sector. At the same time, given that many Tanzanian women continue to work in the informal economy, measures like providing health insurance, skills development, and legal protections are essential to enhance the security and empowerment of women in these roles. Supporting women across both sectors can strengthen their economic resilience and improve overall safety.

## Supplementary Information

Supplementary Material 1.

Supplementary Material 2.

## Data Availability

The datasets used and/or analysed during the current study are available from the corresponding author and Dr. Heidi Stöckl on reasonable request.
