# Supplementary material for: Women’s self-employment, business acumen, and emotional IPV: a longitudinal study in Tanzania
Source: BMC Womens Health. 2026 Mar 5;26:194. doi: 10.1186/s12905-026-04369-3 (PMC13064284; doi:10.1186/s12905-026-04369-3)
Supplement: Supplementary file 1 — Supplementary Material 1. [file 12905_2026_4369_MOESM1_ESM.docx]

Appendix tables:

Table 1 Prevalence Of The Various Finance Acumen Variables In The Subsample Of Self-Employed Women

|  | **Wave** | | | |
| --- | --- | --- | --- | --- |
| **Variable** | **1**, N = 839*^1^* | **2**, N = 715*^1^* | **3**, N = 700*^1^* | **4**, N = 696*^1^* |
| **Ownership of business** | 632 (75.3%) | 555 (77.6%) | 559 (80%) | **565 (80%)^*^** |
| **Leadership of business** | 761 (90.7%) | **629 (88%)^*^** | 641 (92%) | **571 (82%)^*^** |
| **Work hours** |  | | | |
| 0-4 hours | 170 (20.3%) | **112 (15.6%)^*^** | **79 (11%)^*^** | 128 (19%) |
| 5-8 hours | 285 (34%) | **250 (35%)^*^** | **274 (39%)^*^** | 239 (35%) |
| More than 9 hours | 364 (43.4%) | **349 (48.8%)^*^** | **347 (50%)^*^** | 325 (47%) |
| More than 8 hours (binary) | 458 (57.8%) | **432 (60.4%)** | **425 (60.7%)** | 389 (55.9%) |
| **Investment for business** |  | | | |
| None of it | 374 (44.6%) | 371 (51.9%) | 364 (52%) | **337 (48.4%)^*^** |
| Some of if | 219 (26.1%) | 180 (25.2%) | 206 (29.4%) | **234 (33.6%)^*^** |
| Half of it | 69 (8.2%) | 54 (7.6%) | 82 (11.7%) | **57 (8.1%)^*^** |
| Most of it | 29 (3.5%) | 29 (4%) | 38 (5.4%) | **22 (3.1%)^*^** |
| All of it | 10 (1.2%) | 2 (0.3%) | 10 (1.4%) | **3 (0.4%)^*^** |
| Any investment (binary) | 327 (39%) | 265 (37.1) | 336 (48%) | **316 (45.4%)** |
| *^1^*Count (n) or Frequency (%)  Note: *Boldface indicate McNemar’s Χ^2^ test or Wilcox test at p-value <0.05. Wilcox test was used for categorical variables and mcNemar for binary variables. | | | | |

Table 2 Prevalence Of Emotional IPV For The Whole Sample

|  | **Wave** | | | |
| --- | --- | --- | --- | --- |
| **Variable** | **1**, N = 1,004*^1^* | **2**, N = 892*^1^* | **3**, N = 867*^1^* | **4**, N = 836*^1^* |
| **Insult** | 409 (40.73%) | 373 (41.81%) | **389 (44.86%)^*^** | **432 (51.67%)^*^** |
| **Humiliate** | 148 (14.74%) | 155 (17.37%) | **127 (14.64%)^*^** | 111 (13.2%) |
| **Scare** | 175 (17.43%) | 169 (18.94%) | **129 (14.87%)^*^** | 117 (14%) |
| **Threat** | 194 (19.32%) | 182 (20.40%) | **155 (17.88%)^*^** | 136 (16.27%) |
| **Any Emotional IPV** | 449 (44.7%) | 413 (46.3%) | 416 (48%) | **449 (53.7%)^*^** |
| *^1^*Count (n) or Frequency (%)  Note: *Boldface indicate McNemar’s Χ^2^ test at p-value <0.05 compared to wave 1 or baseline. | | | | |

Table 3 Binary Regression Between Composite Emotional IPV And Four Finance Acumen Variables

| **Predictor** | **Current emotional IPV (composite)** | | |
| --- | --- | --- | --- |
|  | **OR1** | **95% CI1** | **p-value** |
| Ownership | 0.75 | 0.59, 0.95 | **0.015** |
| Leadership | 0.63 | 0.47, 0.85 | **0.003** |
| More than eight hours of work | 0.81 | 0.66, 0.99 | **0.038** |
| Any investment | 0.70 | 0.57, 0.85 | **<0.001** |
| 1 OR = Odds Ratio, CI = Confidence Interval. Subsample of self-employed women. | | | |

Table 4 Multivariable Unadjusted Model For Composite Emotional IPV And Finance Acumen Variables

| **Current emotional IPV (composite**) | | | |
| --- | --- | --- | --- |
| **Predictors** | **OR^1^** | **95% CI^1^** | **p-value** |
| Ownership | 0.82 | 0.64, 1.05 | 0.11 |
| Leadership | 0.67 | 0.49, 0.92 | **0.012** |
| More than eight hours of work | 0.79 | 0.64, 0.98 | **0.029** |
| Any investment | 0.69 | 0.56, 0.85 | **<0.001** |
| ^1^OR = Odds Ratio, CI = Confidence Interval Subsample of self-employed women. Dummy variable for Wave is included as a fixed effect. | | | |


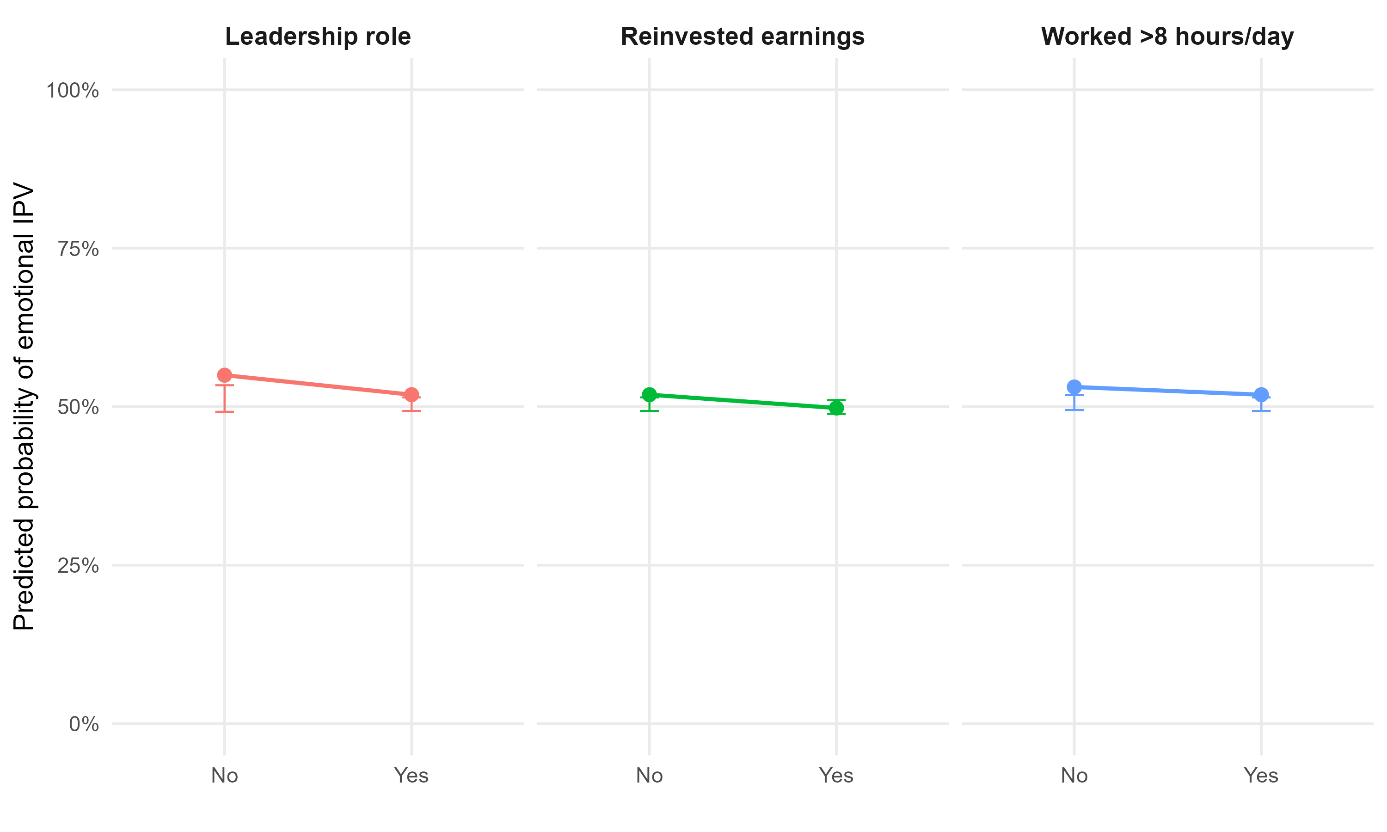


Figure 1 Predicted probability of emotional IPV

Predicted probabilities from the adjusted mixed‑effects model indicated that women without a leadership role had a 55% probability of emotional IPV (95% CI: 0.49–0.53), compared to 52% among women in leadership roles (95% CI: 0.49–0.51). The average marginal effect showed that business leadership was associated with a 9‑percentage‑point reduction in emotional IPV (AME = –0.09, 95% CI: –0.17 to –0.01, p = 0.032).

Women who reinvest any part of their business earnings have a lower predicted probability of experiencing emotional IPV (50%, 95% CI 0.49–0.51) compared to those who do not reinvest (52%, 95% CI 0.49–0.51). The average marginal effect indicates a statistically significant 10‑percentage‑point reduction in predicted IPV risk associated with reinvestment (AME = –0.10, 95% CI –0.15 to –0.05, p < .001), adjusting for sociodemographic covariates and wave.

Predicted probabilities showed minimal differences in emotional IPV between women working ≤8 hours/day (53%, 95% CI 0.49–0.52) and those working >8 hours/day (52%, 95% CI 0.49–0.51). The average marginal effect of long work hours was not statistically significant (AME = –0.04, 95% CI –0.09 to 0.01, p = 0.14), indicating that working more than eight hours per day does not meaningfully change women’s predicted risk of emotional IPV in the adjusted model.

R code files can be found on OSF: <https://osf.io/vyfcx/overview>
