# Supplementary material for: Women’s self-employment, business acumen, and emotional IPV: a longitudinal study in Tanzania
Source: BMC Womens Health. 2026 Mar 5;26:194. doi: 10.1186/s12905-026-04369-3 (PMC13064284; doi:10.1186/s12905-026-04369-3)
Supplement: Supplementary file 2 — Supplementary Material 2. [file 12905_2026_4369_MOESM2_ESM.pdf]

FEMALE PARTICIPANT QUESTIONNAIRE

FOLLOW UP
COMPONENT A
Round 2

CONFIDENTIAL UPON COMPLETION

SECTION 0: ABOUT YOUR HOUSHOLD 4

SECTION 1: ABOUT YOU AND YOUR PARTNER 6

SECTION 2: ABOUT YOU AND YOUR INCOME 10

SECTION 3: ABOUT YOU AND YOUR HEALTH 13

SECTION 4: ABOUT ATTITUDES AND SOCIAL NORMS 16

SECTION 5: ABOUT YOUR RELATIONSHIPS AND CHILD DISCIPLINE 19

SECTION 6: ABOUT YOUR PARTNER'S CHILDHOOD 27

SECTION 7: ABOUT YOUR COMMUNITY 28

Introduction

Hello, my name is _____________________ , I am from the National Institute for Medical Research/Mwanza Interventions Trial Unit. As you know, you have agreed to take part in the study we are currently conducting in Mwanza. One year ago we asked you a number of questions about yourself, your household, your relationship and your community as part of the MAISHA longitudinal study. To better understand changes in your life, we would now like to ask you some similar questions again. This is the last time we will interview you for this study. Some of the questions are sensitive, but everything that you say will be kept private, and will not be shared with others. I would like to encourage you to be honest, as there are no right or wrong answers. At any point you can stop the interview, or choose to not answer a question. If you find anything that we discuss upsetting, and would like to talk to someone afterwards, we can help with this.

The interview will take around two hours to complete. For this, it is best if we are not interrupted. Is this a good place to talk, or should we go somewhere else where we can talk privately?

Do you have any questions?

BEFORE YOU START

**Identification**	
Branch name	
1. Branch code	**[ ][ ][ ][ ][ ][ ]**
2. Group code	**[ ][ ][ ][ ][ ]**
3. Participant no	**[ ][ ]**
**4. P**articipant's ID	**[ ][ ][ ][ ][ ][ ]-[ ][ ][ ][ ][ ]-[ ][ ]**

PLEASE FILL IN COMPLETE PARTICIPANT ID AT THE BOTTOM OF EVERY PAGE OF THIS QUESTIONNAIRE!

Interview details – START

Date of interview: **[ ][ ] /[ ][ ][ ]/ [ ][ ][ ][ ] dd/MMM/yyyy**

Time interview started: **[ ][ ]: [ ][ ]**

Name of interviewer: **[ ][ ][ ]**

SECTION 0: ABOUT YOUR HOUSHOLD

I would like to start by asking you a few questions about your household. When talking of your household, I mean the place and the people that you normally share food with, and sleep with under the same roof.

MAKE SURE THAT ALL QUESTIONS APPLY TO HER HOUSEHOLD AND NOT THE PLACE YOU ARE CURRENTLY CONDUCTING THE INTERVEIW.

	QUESTIONS	CODING CATEGORIES	
000	How many people live in your household and share food with you?<br>PROBE: Does this include children and infants? Does it include any other people who may not be members of your family, such as domestic servants, lodgers or friends who live with you and share food?<br>MAKE SURE THAT ALL OF THESE PEOPLE ARE INCLUDED IN THE TOTAL	TOTAL NUMBER OF PEOPLE IN HOUSEHOLD [ ][ ]	
001	Is the head of the household male or female?	Male<br>Female	1<br>2
002	How long have you been living continuously in Mwanza?	Years: [ ][ ] Months: [ ][ ]	
003	Is the house you live in rented, owned by you (either on your own, or with someone else), or owned by someone else in your family?	Rent<br>Own themselves<br>Owned by someone else in family<br>Owned by someone else other than family member<br>Owned together with someone	1<br>2<br>3<br>4<br>5
004	How many rooms does your household use for sleeping?	[ ][ ]	
005	What type of toilet is mainly used in your household?	Bush<br>Uncovered pit latrine<br>Covered pit latrine shared<br>Covered pit latrine private<br>VIP latrine shared<br>VIP latrine private<br>Flush toilet shared<br>Flush toilet private<br>Other (specify)...............................	1<br>2<br>3<br>4<br>5<br>6<br>7<br>8<br>9
	FOR QUESTIONS 106 TO 108 ANSWER THROUGH OBSERVATION IF POSSIBLE. IF THE INTERVIEW IS TAKING PLACE ELSEWHERE, ASK THE RESPONDENT DIRECTLY.<br>IF THERE IS MORE THAN ONE HOUSE, REFER TO THE MAIN HOUSE.		
006	What is the major construction material of the roof?	Thatch, Straw<br>Mud and poles<br>Tin<br>Wood<br>Iron sheet<br>Tiles<br>Cement	1<br>2<br>3<br>4<br>5<br>6<br>7
007	What is the major construction material of the external wall?	Thatch, Straw<br>Mud and poles<br>Timber<br>Un-burnt bricks<br>Burnt bricks with mud<br>Burnt bricks with cement<br>Cement blocks<br>Stone	1<br>2<br>3<br>4<br>5<br>6<br>7<br>8
008	What is the major construction material of the floor?	Earth<br>Earth and cow dung<br>Cement<br>Mosaic or tiles<br>Stone	1<br>2<br>3<br>4<br>5
009	What is the main source of lighting?	Electricity<br>Paraffin, kerosene or gas lantern<br>Firewood	1<br>2<br>3

		Candle	4
		Other (Specify)……………………………	5
010	What type of fuel do you primarily use for cooking?	Firewood	1
		Charcoal	2
		Paraffin/kerosene	3
		Electricity	4
		Gas	5

		11Respondent		12 Other household member	
	Do **you** or any other household member own any of the following: (READ LIST, CIRCLE ALL THAT APPLY)<br>RESPONDENT REFERS TO HERSELF ONLY. OTHER HOUSEHOLD MEMBER REFERS TO THINGS OWNED BY HUSBAND OR OTHERS TOGETHER				
		Yes	No	Yes	No
a.	Radio	1	2	1	2
b.	Mobile phone	1	2	1	2
c.	Television	1	2	1	2
d.	Fridge	1	2	1	2

		13. Respondent		14 Other household member	
	Do you or any other household member own any of the following:				
		Yes	No	Yes	No
a.	Bicycle	1	2	1	2
b.	Motorcycle/scooter	1	2	1	2
c.	DalaDala (Public Transport)	1	2	1	2
d.	Car	1	2	1	2

		15 Respondent		Other household member 16	
	Do you or any other household member own any of the following:				
		Yes	No	Yes	No
a.	Agricultural Land	1	2	1	2
b.	Large Livestock (cows, pigs, horses)	1	2	1	2
c.	Small animals (goats, chickens, etc)	1	2	1	2
d.	A business or shop	1	2	1	2

SECTION 1: ABOUT YOU AND YOUR PARTNER

I would now like to ask you a few questions about yourself and your partner.
Please remember that everything you mention will be kept confidential and not shared with anyone from your community.

	QUESTIONS	CODING CATEGORIES	
17	How old are you?<br><br>GET RESPONDENT TO ESTIMATE IF DON'T KNOW EXACTLY	Years: [ ][ ]	
18	What is your date of birth?<br>(RECORD AS MUCH INFORMATION AS POSSIBLE. CODE 96 IF DAY UNKNOWN, 996 IF MONTH UNKNOWN, AND 9696 IF YEAR UNKNOWN)	DAY [ ][ ]<br>MONTH (MMM) _ _ _<br>YEAR [ ][ ] [ ][ ]	
21	What is the highest level of schooling you ever completed?	Never went to school<br>Primary incomplete<br>Primary complete<br>Secondary incomplete<br>Secondary (Form I-IV)<br>Secondary (Form V-VI)<br>College training after primary/secondary school and before university<br>University	1<br>2<br>3<br>4<br>5<br>6<br>7<br><br>8

	QUESTIONS	CODING CATEGORIES		SKIP TO
105.	Are you married or currently living with a man, as if married?	YES<br>NO	1<br>2	 107
106.	Is this the same man as when we last interviewed you on this date:__:___:____?	YES<br>NO	1<br>2	108<br>108
107.	Have you been in a relationship with a man during the past 12 months?	Yes<br>No	1<br>2	106<br>120
108.	How long have (had) you been in this relationship?<br>GET TO ESTIMATE IF DON'T KNOW EXACTLY.CODE 00 IF MONTH UNKNOWN	**Years: [ ][ ]**<br>Months: [ ][ ]		
109.	Is (was) your partner married to another woman?	Yes<br>No<br>DON'T KNOW	1<br>2<br>3	 111<br>111
110.	Are (were) you the first, second, ... wife/partner?<br>CODE 96 IF UNKNOWN	Rank: [ ][ ]		
111.	How old is (was) your partner?<br>CODE 96 IF UNKNOWN	Years: [ ][ ]		
114.	What is the highest level of education that he completed?<br><br>CIRCLE ONE	Never went to school<br>Primary incomplete<br>Primary complete<br>Secondary incomplete<br>Secondary (Form I-IV)<br>Secondary (Form V-VI)<br>College training after primary/secondary school and before university<br>University	1<br>2<br>3<br>4<br>5<br>6<br>7<br><br>8	

		Don't know	9	
115.	Has your partner worked during the past 12 months (either employed or self-employed)?	Yes<br>No	1<br>2	 120
116.	Is this work self-employed or is he employed?	Self employed<br>Employed	1<br>2	
117.	Does your partner usually work throughout the year, or does he work seasonally, or only once in a while?	Throughout the year<br>Seasonally/Part of the Year<br>Once in a while	1<br>2<br>3	
118.	Does your partner have to travel away from home for his work? How long would you say he has been away in the past year (12 months)?:<br>READ RESPONSE CODES AND CIRCLE ONE	More than half of the year<br>About half of the year<br>Less than half of the year<br>Not all	1<br>2<br>3<br>4	
119.	Over a typical month/week in the past year, how much money does your partner earn?<br>USE 969696 IF DON'T KNOW	Month TSH (estimate):[ ][ ] [ ][ ] [ ][ ]<br>Week TSH (estimate): [ ][ ] [ ][ ] [ ][ ]		
**119a.**	When we interviewed you **one** year ago (date; __:___:__) was your partner working for money at that time for the last 12 months?	Yes<br>No<br>I had no partner at that time	1<br>2<br>3	 <br>120
**119b.**	Was he self-employed or employed at that time?	Self employed<br>Employed	1<br>2	
**119c.**	When we interviewed you **two** years ago (date; __:___:__) was your partner working for money at that time for the last 12 months?	Yes<br>No<br>I had no partner at that time	1<br>2<br>3	 <br>121
**119d.**	Was he self-employed or employed at that time?	Self employed<br>Employed	1<br>2	

I would like to learn a bit more about the children that you are responsible for, including both your own children and other children that live with you, as well as children living elsewhere. Please remember this information is confidential and when we are using initials of your children it is only to make sure which child we are talking about. This cannot be used to identify your children!

USE THIS SECTION TO BUILD A RAPPORT WITH THE INTERVIEWEE.

120. Are there children less than 18 years whom you are responsible for? These may include those living in your household or living elsewhere.

1. Yes
2. No (Go to Question 200)

121.	Since we interviewed you on this date; __:__:___, have you given birth or accepted a new child into your household that you are responsible for?		Yes (go to 122)<br>No (go to 128)					
122-127	I will ask you a little about each new child in your household in turn, starting with the eldest child.	START WITH OLDEST.<br>No of child (1-6)	1	12	12	12	126.	127.
a.	What are their initials or nickname?	FILL IN **ALL** INITIALS						
b.	Is this a girl or a boy?	Girl<br>Boy	1<br>2	1<br>2	1<br>2	1<br>2	1<br>2	1<br>2
c.	What is their date of birth?	Day<br><br>Month (MMM)	[ ] [ ]<br><br>_ _ _	[ ] [ ]<br><br>_ _ _	[ ] [ ]<br><br>_ _ _	[ ] [ ]<br><br>_ _ _	[ ] [ ]<br><br>_ _ _	[ ] [ ]<br><br>_ _ _

		Year<br><br>(USE 96 FOR ALL IF NOT KNOWN)	[] [] [] []	[] [] [] []	[] [] [] []	[] [] [] []	[] [] [] []	[] [] [] []
d.	How old is he/she?	IF DATE UNKNOWN USE 96.<br>USE 00 IF <1 YEAR	[ ] [ ]	[ ] [ ]	[ ] [ ]	[ ] [ ]	[ ] [ ]	[ ] [ ]
e.	Is [NAME/initials] living with you?	No - in another household<br>No - away at school<br>Yes	1<br>2<br>3	1<br>2<br>3	1<br>2<br>3	1<br>2<br>3	1<br>2<br>3	1<br>2<br>3
f.	Are you the mother?	Yes<br>No - Other woman	1<br>2	1<br>2	1<br>2	1<br>2	1<br>2	1<br>2
g.	Is the father your current partner, or another man?	Current partner<br>Previous partner<br>Other man	1<br>2<br>3	1<br>2<br>3	1<br>2<br>3	1<br>2<br>3	1<br>2<br>3	1<br>2<br>3
h.	Is [NAME/initials] at school?	Never been (SKIP to J)<br>In the past<br>Currently	1<br>2<br>3	1<br>2<br>3	1<br>2<br>3	1<br>2<br>3	1<br>2<br>3	1<br>2<br>3
i.	What is the highest grade they reached?	Primary<br>Secondary<br>Nursery<br>college	1<br>2<br>3<br>4	1<br>2<br>3<br>4	1<br>2<br>3<br>4	1<br>2<br>3<br>4	1<br>2<br>3<br>4	1<br>2<br>3<br>4
j.	IF NOT ENROLLED IN SCHOOL: What is the <u>main</u> reason why they aren't in school? [NB do not read out answers]	Truancy<br>Pregnancy<br>Marriage<br>School fees<br>Lack of money<br>Illness of child<br>Caring for family members<br>Failed exams<br>Farming<br>Household chores<br>Too young<br>Other (Specify)	1<br>2<br>3<br>4<br>5<br>6<br>7<br>8<br>9<br>10<br>11<br>12	1<br>2<br>3<br>4<br>5<br>6<br>7<br>8<br>9<br>10<br>11<br>12	1<br>2<br>3<br>4<br>5<br>6<br>7<br>8<br>9<br>10<br>11<br>12	1<br>2<br>3<br>4<br>5<br>6<br>7<br>8<br>9<br>10<br>11<br>12	1<br>2<br>3<br>4<br>5<br>6<br>7<br>8<br>9<br>10<br>11<br>12	1<br>2<br>3<br>4<br>5<br>6<br>7<br>8<br>9<br>10<br>11<br>12
k.	IF HAVE LEFT SCHOOL: What age did they leave at?		[ ] [ ]	[ ] [ ]	[ ] [ ]	[ ] [ ]	[ ] [ ]	[ ] [ ]
l.	IF STILL IN SCHOOL: Are they currently repeating year?	Yes<br>No<br>Don't know	1<br>2<br>3	1<br>2<br>3	1<br>2<br>3	1<br>2<br>3	1<br>2<br>3	1<br>2<br>3

128.	Do you have a child aged 5-12 years living in your household?<br>If yes, does any of these children:	Yes<br>No	1<br>2	 128x
a.	Have frequent nightmares?	Yes<br>No	1<br>2	
b.	Suck their thumbs or fingers?	Yes<br>No	1<br>2	
c.	Often wet their bed?	Yes<br>No	1<br>2	
d.	Are any of these children very timid or withdrawn?	Yes<br>No	1<br>2	
e.	Are any of them aggressive with you or other children?	Yes<br>No	1<br>2	

128x.	Do you have one or more teenage children aged 13-18 years living in your household?	Yes<br>No	1<br>2	 Section 2 (200)
A	Is any of your teenage children attending secondary school?	Yes<br>No	1<br>2	Ask Ba<br>Ask C
Ba	Has any of your teenage children missed more than 2 weeks of school in the last term?	Yes<br>No	1<br>2	
Bb	Has any of your teenage children ever repeated a grade or failed their last grade?	Yes<br>No	1<br>2	
C	Is any of your teenage children currently employed or working?	Yes<br>No	1<br>2	
D	Has any of your teenage children received career development or advice through groups, school or other organizations?	Yes<br>No	1<br>2	
E	Is any of your teenage children married or living as if married with a man/woman?	Yes<br>No<br>Refuse to answer	1<br>2<br>3	
F	Is any of your teenage children pregnant/has given birth to a child or has fathered a child (if a boy)?	Yes<br>No	1<br>2	
G	Has any of your teenage children ever had TB or shown likely symptoms of undiagnosed TB?	Yes<br>No	1<br>2	
H	Is any of your teenage children living with HIV/AIDS?	Yes<br>No<br>Don't know		
I	Has any of your teenage children access to a mobile phone?	Yes<br>No	1<br>2	
J	Did you ever participate in any parenting programme for example in a church or mosque? Or receive home visits from someone who discussed parenting?	Yes<br>No	1<br>2	
K	Did you send any of your teenage children to a creche or preschool?	Yes<br>No	1<br>2	

SECTION 2: ABOUT YOU AND YOUR INCOME

I would now like to find out a bit more about the ways that you earn money.

200.	What are the main sources of income for you and your family? CAN ALLOW MULTIPLE RESPONSES	Yes	No	
a.	Money from own work	1	2	
b.	Support from husband/partner	1	2	
c.	Support from other relatives	1	2	
d.	Pension	1	2	
e.	Social services/welfare	1	2	
f.	Salary from an employer	1	2	
g.	Other (specify) ………………………	1	2	

201.	Have you personally earned money during the past 12 months?	Yes<br>No	1<br>2	 217
202.	Are you self-employed or do you work for someone else/organization?<br><br>IF WORKING FOR SOMEONE ELSE OR AN ORGANIZATION CONTINUE UNTIL **209** THEN SKIP TO **217**	Self employed<br>Worked for someone else or an organization<br>Both<br>Not employed at the moment	1<br>2<br>3<br>4	210<br><br><br>210
203.	(IF WORKING FOR SOMEONE ELSE OR AN ORGANISATION),<br>Who is your main employer?	Relative<br>Neighbor<br>Friend / acquaintance<br>Government<br>NGO<br>Private company / Someone's business<br>Other (Specify):........	1<br>2<br>3<br>4<br>5<br>6<br>7	
The following questions refer only to work you did while employed by someone else or an organization<br>Skip if not employed by someone else				
204.	Is this a regular or an occasional activity?	Regular<br>Occasional	1<br>2	 206
205.	Do you usually work throughout the year, or do you work seasonally, or only once in a while?	Throughout the year<br>Seasonally/Part Of The Year<br>Once in a while	1<br>2<br>3	
206.	For how many months in total did you do this activity during the past year?	1 month or less<br>1-6 months<br>6-9 months<br>9-12 months	1<br>2<br>3<br>4	
207.	Are you paid in cash, or given other items such as food?	Cash only<br>Cash and items<br>Items only	1<br>2<br>3	 <br>209
208.	On a typical working day/week/month how much do you earn from this activity?<br>FILL OUT ONLY ONE, EITHER DAY, WEEK OR MONTH. ENTER 969696 IF DON'T KNOW	Day: [ ] [ ] [] [ ] [] [ ]<br>Week: [ ] [ ] [] [ ] [] [ ]<br>Month: [ ] [ ] [] [ ] [] [ ]		
209.	Do you plan to continue this activity over the next 12 months?	Yes<br>No<br>Don't know	1<br>2<br>96	
	If self-employed...			

			Yes	No
210.	What kind of business do you run? (MULTIPLE RESPONSES OK)	a. Selling vegetables	1	2
		b. Selling homemade food	1	2
		c. Running small restaurant	1	2
		d. Sewing or fixing cloths	1	2
		e. Selling cloths	1	2
		f. Brewing/Selling Alcohol	1	2
		g. Running a salon	1	2
		h. Running a shop	1	2
		I. Selling fish	1	2
		j. Other (Specify):………………………	1	2

211.	Have you been operating this business (main) for more than or less than 12 months?	Less than 12 months<br>More than 12 months	1<br>2	
212.	Are you mainly responsible for this activity, or are others responsible?	I am mainly responsible<br>Others are responsible	1<br>2	
213.	On a typical working day, how many hours do you work?<br>ENTER 96 IF DON'T KNOW	Hours: [ ] [ ]		
214.	During the past month, how many times did you not earn enough to cover the costs of running your business?	Never<br>Once<br>Few times<br>Many times<br>Not working at the time being	1<br>2<br>3<br>4<br>5	
215.	Do you employ other people and pay them a wage?	Yes<br>No	1<br>2	
216.	Of the money you earned over the past month, what proportion did you reinvest to maintain or expand your business?	None of it<br>Some of if<br>Half of it<br>Most of it<br>All of it<br>Not working at the time being	1<br>2<br>3<br>4<br>5<br>6	

	QUESTIONS	CODING CATEGO RIES		SKIP TO
	I would now like to ask you some questions about whether you have been using a microfinance provider, such as SACCOS, BRAC, FINCA or others?			
217.	In the past 12 months, have you ever borrowed money from a microfinance provider?	Yes<br>No	1<br>2	 224
218.	Are you still borrowing money from one of these organization?	Yes<br>No	1<br>2	
219.	How much money did you borrow?	Amount [ ] [ ] [ ] [ ] [ ] [ ] [ ] [ ]		
220.	How do/did you **primarily** use the money you borrow?<br><br>READ OUT RESPONSES	To build a small business<br>To maintain a business<br>To pay for medical expenses<br>To pay for school expenses<br>To buy food or clothes<br>To pay back another microfinance loan<br>To pay for rent<br>To help other family members<br>Others (specify)	1<br>2<br>3<br>4<br>5<br>6<br>7<br>8<br>9	
221.	How confident do you feel about being able to pay this back? Would you say that you feel:<br>Very confident<br>Somewhat confident<br>Not very confident?	Very confident<br>Somewhat confident<br>Not very confident	1<br>2<br>3	
222.	How important is the money that you get from microfinance groups? Would you say that:<br>It is extremely important<br>Somewhat important<br>Not very important at all?	Extremely important<br>Somewhat important<br>Not very important	1<br>2<br>3	
223.	In the past 12 months, overall, how has you being a member of microfinance groups influenced your relationship with your partner? Would you say that it has:<br>Had no effect on the relationship<br>Made your relationship more difficult<br>Improved your relationship?	Not effected the relationship<br>Made your relationship more difficult<br>Improved your relationship<br>Not in a relationship	1<br>2<br><br>3<br>9	

	QUESTIONS	CODING CATEGORIES			
224.	Nowadays, many families have a hard time making ends meet. I would like to learn more about how your household is coping. During the last 12 months, how many times…<br><br>EMPHASIZE THAT YOU ARE TALKING ABOUT THE PAST **12 MONTHS**<br><br>**In the past 12 months…….**		If **notNEVER**…		
		Never	Once	Few times	Many times
a.	… were you very worried/stressed about your general financial situation. Would you say, this has happened or never happened?	1	2	3	4
b.	… have you had trouble buying food or other necessities for your family? Would you say, this has happened or never happened?	1	2	3	4
c.	… have you had to borrow money to pay rent or other bills ? Would you say, this has happened or never happened?	1	2	3	4
d.	… did any of your family members need to see a doctor/go to a health facility but could not because you did not have enough money? Would you say, this has happened or never happened?	1	2	3	4
e.	… did your children miss days of school because you did not have money for school fees, uniforms or supplies? Would you say, this has happened or never happened?	1	2	3	4
f.	… have you or any of your own children gone a whole day without eating anything because there was not enough food? Would you say, this has happened or never happened?	1	2	3	4

	QUESTIONS	CODING CATEGORIES			QUESTIONS	CODING CATEGORIES	
225.	During the past 12 months, how important is the money that you yourself bring into the family? Would you say that it is extremely important, somewhat important, or not very important at all?	Extremely important<br>Very important<br>Somewhat important<br>Not very important<br>Don't bring in any money	1<br>2<br>3<br>4<br>5	226.	During the past 12 months, how important does your partner think is the money that you yourself bring into the family?<br>Would you say he thinks it is extremely important, somewhat important, or not very important at all?	Extremely important<br>Very important<br>Somewhat important<br>Not very important<br>Don't bring in any money<br>Has no partner<br>Don't know	1<br>2<br>3<br>4<br>5<br>6<br>7
227.	Would you say that the money that you bring into the household is more than what your husband/partner contributes, less than what he contributes, or about the same as he contributes?	Less than partner<br>Same as Partner<br>More than partner<br>Has no partner	1<br>2<br>3<br>4	228.	Would your partner say that the money that you bring into the household is more than what he contributes, less than what he contributes, or about the same as he contributes?	Less than partner<br>Same as Partner<br>More than partner<br>Has no partner<br>Don't know	1<br>2<br>3<br>4<br>5
229.	Do you think that you would be able to look after yourself and your family on your income alone? Would you say Definitely yes, yes with difficulty, probably not, or definitely not?	Yes definitely<br>Yes with difficulty<br>Probably not<br>Definitely not	1<br>2<br>3<br>4	230.	Would your partner think that you would be able to look after yourself and your family on your income alone?<br>Would you say Definitely yes, yes with difficulty, probably not, or definitely not?	Yes, definitely<br>Yes, with difficulty<br>Probably not<br>Definitely not<br>Has no partner<br>Don't know	1<br>2<br>3<br>4<br>5<br>6

231a.	What expenses do you contribute in your household?	Food<br>School fees<br>School bus-fare/school pocket money<br>Children's clothes<br>Treatment<br>House rent<br>House bills e.g. water<br>Development projects (house)<br>Household expenses e.g. housekeeping<br>No contribution<br>Other (Specify:………………)	1<br>2<br>3<br>4<br>5<br>6<br>7<br>8<br>9<br>10<br>11	231b.	What expenses does your partner contribute in your household?	Food<br>School fees<br>School bus-fare/school pocket money<br>Children clothes<br>Treatment<br>House rent<br>House bills e.g. water<br>Development projects (house)<br>Household expenses e.g. house-keeping<br>No partner<br>No contribution<br>Other (Specify:………..)	1<br>2<br>3<br>4<br>5<br>6<br>7<br>8<br>9<br>10<br>11<br>12
231c.	How satisfied are you with your contribution?	Very satisfied<br>Satisfied<br>More or less satisfied<br>Somewhat unsatisfied<br>Very unsatisfied	1<br>2<br>3<br>4<br>5	231d.	How satisfied are you with your partner's contribution?	Very satisfied<br>Satisfied<br>More or less satisfied<br>Somewhat unsatisfied<br>Very unsatisfied<br>No partner	1<br>2<br>3<br>4<br>5<br>6

SECTION 3: ABOUT YOU AND YOUR HEALTH

300	I would like to learn about your approach to life, in general. I am going to read you some statements, and I would like you to say whether they are true or false:<br>If true,<br>Ask somewhat true, true or definitely true<br>If not true,<br>Ask, somewhat false, false, or definitely false	Definitely true	Mostly true	Slightly true	Slightly false	Mostly false	Definitely f false
a.	You cannot think of many ways to get out of a difficult situation when life evolves unexpectedly.	1	2	3	4	5	6
b.	You energetically pursue your goals.	1	2	3	4	5	6
c.	You feel tired most of the time.	1	2	3	4	5	6
d.	There are lots of ways around any problem.	1	2	3	4	5	6
e.	You are easily downed in an argument.	1	2	3	4	5	6
f.	You cannot think of many ways to get the things in life that are important to you.	1	2	3	4	5	6
g.	You worry about your health.	1	2	3	4	5	6
h.	Even when others get discouraged, you know you can find a way to solve the problem.	1	2	3	4	5	6
i.	Your past experiences have not prepared you well for your future.	1	2	3	4	5	6
j.	You've been pretty successful in life.	1	2	3	4	5	6
k.	You usually find yourself worrying about something.	1	2	3	4	5	6
l.	You do not meet the goals that you set for yourself.	1	2	3	4	5	6

I would now like to ask you some questions about your health.

301	During the last 4 weeks, have you been bothered by any of the following problems?	Yes	No
a.	Do you often have headaches?	1	2
b.	Is your appetite poor?	1	2
c.	Do you sleep badly? Like difficulties falling asleep, waking up in the middle of the night more than 3 times or waking up early in the morning and not getting back to sleep.	1	2
d.	Are you easily frightened?	1	2
e.	Do your hands shake?	1	2

f.	Do you feel nervous, tense or worried?	1	2
g.	Is your digestion poor? Like you are often constipated, feel nauseous, or you don't have an appetite.	1	2
h.	Do you have trouble thinking clearly?	1	2
i.	Do you feel unhappy?	1	2
j.	Do you cry more than usual? Like every day or more than once per day, because of problems?	1	2
k.	Do you find it difficult to enjoy your daily activities?	1	2
l.	Do you find it difficult to make decisions?	1	2
m.	Is your daily work suffering?	1	2
n.	Are you unable to play a useful part in life?	1	2
o.	Have you lost interest in things?	1	2
p.	Do you feel that you are a worthless person?	1	2
q.	Has the thought of ending your life been on your mind?	1	2
r.	Do you have uncomfortable feelings in your stomach?	1	2
s.	Are you easily tired?	1	2

302	Introductory phrase: The next questions ask about difficulties you may have doing certain activities because of a HEALTH PROBLEM.	No – no difficulty	Yes- some difficulty	Yes – a lot of difficulty	Cannot do at all
a.	Do you have difficulty seeing, even if wearing glasses	1	2	3	4
b.	Do you have difficulty hearing, even if using a hearing aid?	1	2	3	4
c.	Do you have difficulty walking or climbing steps?	1	2	3	4
d.	Do you have difficulty remembering or concentrating?	1	2	3	4
e.	Do you have difficulty (with self-care such as) washing all over or dressing?	1	2	3	4
f.	Using your usual (customary) language, do you have difficulty communicating, for example understanding or being understood?	1	2	3	4
302x	"Do you have any long-standing illness, disability or infirmity?<br>By long-standing I mean anything that has troubled you over a period of time, or that is likely to affect you over a period of time?"	1<br>2	No<br>Yes (Specify :………..)	If Yes 302xa if No 303	
		No –limitation	Yes-some limitation	Yes-a lot of limitation	Yes-full limitation
302xa	Has the long-standing illness, disability or infirmity limited your activities in any way?	1	2	3	4
302xb	Where did you seek help when you experienced these conditions?	1	Health facility		
		2	Church/Mosque		
		3	Traditional healer		
		4	Did not seek help		
		5	Other: (Specify:…………)		

I would now like to ask you some questions about your reproductive health. Some of the questions may be embarrassing to

	answer. Please remember that this information is confidential and you may choose not to answer them.			
303<br><br>304	Are you currently or have you been pregnant since the last interview?<br>If YES, how many times have you been pregnant since we last interviewed you?	Yes<br>No<br>No response<br>[ ]	1<br>2<br>3	 305<br>305
305	Are you currently using modern contraceptive methods like hormonal contraceptives, loop or alike?	Yes<br>No<br>Pregnant	1<br>2<br>3	
306	Would you describe your sexual relationship with your main partner as:	Very satisfying<br>Satisfying<br>More or less satisfying<br>Somewhat unsatisfying<br>Very unsatisfying<br>Has no partner<br>No response<br><br>(IF SHE HAS NO PARTNER SKIP TO **310)**	1<br>2<br>3<br>4<br>5<br>6<br>7	
307	How many people in total have you had sexual intercourse with in the past year?<br>CODE 99 IF HAS REFUSED TO ANSWER.<br>IF 00 SKIP TO QN 310	Give total number:	[ ][ ]	
308	During the last 12 months, have you ever received money or material goods in exchange for sex?	Yes<br>No<br>No response	1<br>2<br>99	
309	In the past 12 months, when you had sex, how often did you use a condom? Would you say:<br>READ RESPONSES	Almost always<br>Most of the time<br>Some times<br>Almost never<br>Not having sexual intercourse	1<br>2<br>3<br>4<br>5	
310	In the past 12 months, *have you* had an HIV test?	Yes<br>No<br>No response	1<br>2<br>99	If Yes go to 310x<br><br>If No go to 311
310x	If you don't mind, could you tell me the result; whether positive or negative HIV test?	Yes (Positive)<br>No (Negative)<br>No response	1<br>2<br>99	
	Increasingly women and men in Tanzania are drinking alcoholic beverages. If you don't mind, I would like to ask you about yours and your husband/partner's use of alcohol. Please feel free to be open.			
311	In the past 12 months, have you ever drunk an alcohol-containing beverage? For example beer, wine, local brew, local spirit (eg.Gongo) or other alcoholic beverage?	Yes<br>No	1<br>2	 315
312	How often do you have a drink containing alcohol? Would you say:<br>READ RESPONSES:	1 -6 times per year<br>2-4 times a month<br>2-3 times a week<br>4 or more times a week	1<br>2<br>3<br>4	
313	On average, how many drinks containing alcohol do you have on a typical day when you are drinking?	1 or 2<br>3 or 4<br>5 or 6<br>7, 8 or 9<br>10 or more	1<br>2<br>3<br>4<br>5	

		No	Once per year	Once per every month	Once per every week	Daily, almost daily
314	The following questions probe about the number of drinks you normally take AND the number of times you have been able or unable to do routine activities.					
a.	Do you use six or more drinks on one occasion?<br>IF YES , READ OUT ANSWERS	1	2	3	4	5

b.	In the past 12 months, have you found that you were not able to stop drinking once you had started? IF YES, READ OUT ANSWERS	1	2	3	4	5	
c.	In the past 12 months, have you failed to do what was normally expected of you because of drinking? IF YES, READ OUT ANSWERS	1	2	3	4	5	
d.	In the past 12 months, have you needed a first drink in the morning to get yourself going after a heavy drinking session? IF YES , READ OUT ANSWERS	1	2	3	4	5	
e.	In the past 12 months, have you had a feeling of guilt or remorse after drinking? IF YES , READ OUT ANSWERS	1	2	3	4	5	
f.	In the past 12 months, have you been unable to remember what happened the night before because of your drinking? IF YES , READ OUT ANSWERS	1	2	3	4	5	

g.	Have you or someone else been injured because of your drinking – either in the past 12 months, or before this?	Yes, during the past 12 months<br>Yes, but not in the last year<br>No	1<br>2<br>3	
h.	Has a relative, friend, doctor, or other health care worker been concerned about your drinking or suggested you cut down?	Yes, during the past 12 months<br>Yes, but not in the last year<br>No	1<br>2<br>3	
315	Does your partner drink alcohol?	Yes<br>No<br>No partner	1<br>2<br>3	 320<br>Section 4
316	In the past 12 months, how often have you seen your partner intoxicated (drunk)?	Never<br>Once<br>Few times<br>Many times	1<br>2<br>3<br>4	
317	In the past 12 months, has he ever failed to do what was normally expected of him because of drinking?	Yes<br>No	1<br>2	
318	In the past 12 months, has your partner injured himself or someone else because of his drinking?	Yes<br>No	1<br>2	
319	In the past 12 months, were you ever concerned about your partner's drinking such that you would suggest a cut down?	Yes<br>No	1<br>2	
In the following questions, we would like to know more about your partner's life outside home				
320	Where does your partner spend most of his time with outside work?	Family<br>Friends<br>Mosque/Church<br>Community<br>Other, please specify….	1<br>2<br>3<br>4<br>5	
321	In the past 12 months, how often has your partner been in a physically fight with other people you are not living with?	Never<br>Once<br>Few times<br>Many times	1<br>2<br>3<br>4	
322	Has your partner ever been arrested?	Yes<br>No<br>Don't know	1<br>2<br>3	

SECTION 4: ABOUT ATTITUDES AND SOCIAL NORMS

In this community and elsewhere, people have different ideas about families and what is acceptable behavior for men and women in the home. We would like to know your views on what is acceptable and what you think other people in your community believe.

	QUESTIONS	CODING CATEGORIES

		In your personal opinion…			
400	I am going to make a number of statements about men and women in general. When I read the following statements can you please indicate how much you personally agree or disagree?<br><br>READ STATEMENTS, THEN ASK IF AGREE OR DISAGREE. THEN ASK IF AGREE OR STRONGLY AGREE OR DISAGREE OR STRONGLY DISAGREE	I strongly agree	I agree	I disagree	I strongly disagree
a.	It's a wife's obligation to have sex with her husband even if she doesn't want to.	1	2	3	4
b.	It **must** be the man who is the primary provider for the family.	1	2	3	4
c.	Women should have the same right as men to study and to work outside of the home	1	2	3	4
d.	A woman should obey her husband's wishes even if she disagrees.	1	2	3	4
e.	Even healthy relationships can include hitting each other as long as the partners love each other.	1	2	3	4
f.	It is perfectly acceptable for women to work outside the home to help support the family economically.	1	2	3	4
g.	The leadership of a community should be largely in the hands of men.	1	2	3	4
h.	Children and men would benefit, if fathers were more involved in caring for their children.	1	2	3	4
i.	It is natural and right that men have more power than woman in the family.	1	2	3	4
j.	Women could take on many of the roles of men, if men were willing to share power.	1	2	3	4

		In my personal opinion…			
401	People have different opinions about whether there are situations where a man can be violent towards his partner. When I read the following statements can you please say whether you personally AGREE OR DISAGREE,<br><br>PROBE WHERE NEEDED.<br><br>READ SENTENCE AND ASK IF AGREE , OR STRONGLY AGREE,<br>IF DISAGREE, THEN ASK IF DISAGREE or STRONGLY DISAGREE	I strongly agree	I agree	I disagree	I strongly disagree
a.	A man has a good reason to hit his wife if **she** does not complete her household work to his satisfaction.	1	2	3	4
b.	A man has a good reason to hit his wife if **she** disobeys him.	1	2	3	4
c.	A man has good reason to hit his wife if **she** refuses to have sexual intercourse with him.				
d.	A man does not have any reason to hit his wife in any way.	1	2	3	4
e.	A man has a good reason to hit his wife if **she** protests because he has other girlfriends.	1	2	3	4
f.	A man has a good reason to hit his wife if **he** suspects that she is unfaithful in marriage.	1	2	3	4
g.	A man has a good reason to hit his wife if he finds out that she has been unfaithful in marriage.	1	2	3	4
h.	It is violence if a woman refuses to have sex with her partner without a good reason	1	2	3	4

i.	It is violence if a man refuses to have sex with his partner without a good reason	1	2	3	4
j.	It is violence if a male partner has an affair with another woman	1	2	3	4
k.	It is violence if a female partner has an affair with another man	1	2	3	4
l.	It is violence if a male partner wants to have anal sex with his partner	1	2	3	4
m.	It is violence if a male partner wants to have oral sex with his partner	1	2	3	4

SECTION 5: ABOUT YOUR RELATIONSHIPS AND CHILD DISCIPLINE

PLEASE LOOK AT QUESTION 107. IF THE RESPONSE IS NO (NO PARTNER IN THE PAST 12 MONTHS) AND THE RESPONSE TO QUESTION 120 IS YES SKIP TO QUESTION 533.

IF THE RESPONSES TO BOTH QUESTIONS 107 AND 120 ARE NOGO TO SECTION 7.

When two people marry, live together or are in a relationship, they usually share both good and bad moments. I would now like to ask you some questions about your current and past relationships and how your husband/partner treats (treated) you. If anyone interrupts us I will change the topic of conversation. I would again like to assure you that your answers will be kept secret, and that you do not have to answer any question that you do not want to. May I continue?

	QUESTIONS	CODING CATEGORIES			
500.	During the last 12 months, did you and your partner discuss the following topics together	If happed fill the answer in the shaded area			
		Never	Once	Few times	Many times
a.	... things that happened to you during the day? Would you say never, once, a few times, or many times?	1	2	3	4
b.	... things that happened to him in the day?<br>Would you say never, once, a few times, or many times?	1	2	3	4
c.	... your worries or feelings?<br>Would you say never, once, a few times, or many times?	1	2	3	4
d.	... his worries or feelings?.<br>Would you say never, once, a few times, or many times?	1	2	3	4
501.	During the last 12 month, did your partner ever...	If yes, how often?			
a.	... ask you for your advice to resolve a problem he was facing?<br>Would you say never, once, a few times, or many times?	1	2	3	4
b.	... followed your advice to resolve a problem he was facing?<br>Would you say never, once, a few times, or many times?	1	2	3	4
c.	... helped you in finding work?<br>Would you say never, once, a few times, or many times?	1	2	3	4
d.	... encouraged you to participate in something outside of the home that was only for *your* benefit?<br>Would you say never, once, a few times, or many times?	1	2	3	4
e.	... made you feel appreciated.<br>Would you say never, once, a few times, or many times?	1	2	3	4

502.	How confident do you feel to assert your own opinion if it is different from that of your husband/partner?	Very confident<br>Confident but would need to be encouraged<br>Not confident at all<br>Don't know	1<br>2<br><br>3<br>96
503.	Have you asserted your opinion in the past 12 months?	No<br>Yes	2<br>1
504.	How comfortable would you feel in resisting efforts by your husband/partner to control aspects of your life like who you see and how you spend your money?<br>READ ANSWERS	Very comfortable<br>Comfortable but would need encouragement<br>Not comfortable at all<br>Don't know	1<br>2<br><br>3<br>96

505.	No matter how well a couple gets along, there are times when they disagree. **In your relationship with your current / most recent partner....**	Yes	No		
a.	... would you say that you quarreled in the past 12 months? (SKIP TO QUESTION 507 IF ANSWER IS NO)	1	2		
	How often have you quarreled about: IF HAPPENED, HAS HAPPENED ONCE, FEW TIMES OR MANY TIMES?	Never	Once	Few times	Many times
b.	Accusations that you are not fulfilling your responsibilities as wife and mother	1	2	3	4
c.	His inability or unwillingness to provide for the family	1	2	3	4
d.	Other issues around money and division of resources in the family	1	2	3	4
e.	His drinking/gambling or drug use	1	2	3	4
f.	Your drinking	1	2	3	4
g.	Concerns about outside partners or accusations of infidelity	1	2	3	4
h.	Your refusal to have sex	1	2	3	4
i.	Other issues around sex (frequency, condom use, etc)	1	2	3	4
j.	You disobeying your partner or treating him disrespectfully.	1	2	3	4
k.	Him treating you or your children disrespectfully.	1	2	3	4
l.	Food that you prepared or did not prepare	1	2	3	4
m.	Your business or money-generating activity	1	2	3	4

506.	Now I will ask you about the steps you took after quarreling with your partner.<br><br>In the past 12 months when you have argued with your partner, how generally did you react?<br><br>Would you say that you did the following activities never, once, a few times or many times:	If happed fill the answer in the shaded area			
		Never	Once	Few times	Many times
a.	Expressed how you felt in a calm and respectful way.	1	2	3	4
b.	Tried to see your partner's side and listened carefully to what he had to say.	1	2	3	4
c.	You brought in or tried to bring in someone to help settle things.	1	2	3	4
d.	When you felt the argument got too heated, you left so that you and your partner had time to calm down.	1	2	3	4
e.	You started to make complaints about things unrelated to your initial argument.	1	2	3	4
f.	You insulted or swore at him.	1	2	3	4
g.	You yelled.	1	2	3	4
h.	You pushed, shook or pulled him.	1	2	3	4

507	I am now going to ask you about some situations that are true for many women. Thinking about your (current or most recent or past) husband/partner, would you say it is generally true that he:	507.	507x. ONLY ASK IF "YES" IN 507 Has this happened in the **past 12 months**?		
		Yes	No	Yes	No
a.	Has ever tried to keep you from seeing your friends	1	2	1	2
b.	Has ever tried to restrict contact with your family of birth	1	2	1	2
c.	Has ever Insisted on knowing where you are at all times	1	2	1	2
d.	Has ever been jealous and angry if you spoke with another man	1	2	1	2
e.	Has been suspicious of your faithfulness	1	2	1	2
f.	Has constantly checked your cell phone	1	2	1	2
**508**	Thinking about your (current or most recent/past) husband/partner, would you say it is generally true that he:	508.	508x. ONLY ASK IF "YES" IN 508 Has this happened in the past 12 months		

a.	Has ever refused to give you enough money for household expenses, even when he had money for other things?	1	2	1	2
b.	Has ever taken money that you have earned away from you	1	2	1	2
c.	Has ever made important financial decisions without consulting you	1	2	1	2

509 – 510	The next questions are about things that happen to many women, and that your current partner, or any other partner may have done to you.<br><br>Has your <u>current</u> or last husband/partner **ever** ….	**509** **(If YES continue with 509x If NO go to 509 next sub question, if ALL NO skip to 512)**		509x. Has this happened in the past 12 months? (If YES ask 510. If NO ask 512)		**510** <u>In the past 12 months</u> would you say that this has happened once, a few times or many times?		
		YES	NO	YES	NO	One	Few	Many
a.	Insulted you or made you feel bad about yourself?	1	2	1	2	1	2	3
b.	Belittled or humiliated you in front of other people?	1	2	1	2	1	2	3
c.	Done things to scare or intimidate you on purpose (e.g. by the way he looked at you, by yelling and smashing things)?	1	2	1	2	1	2	3
d.	Verbally threatened to hurt you or someone you care about?	1	2	1	2	1	2	3

**511**	SKIP IF 509 ALL NO<br>Was the person who insulted, humiliated, scared or threatened you your current or a previous partner	Current<br>Previous<br>Both	1<br>2<br>3

512 - 513	Has <u>your current partner or any other partner **ever**</u>…	**512** **(If YES continue with 512x If NO go next 512 sub question if ALL NO skip to 515)**		512x. Has this happened in the past 12 months? (If YES ask 513. If NO ask 615)		**513** <u>In the past 12 months</u> would you say that this has happened once, a few times or many times?		
		YES	NO	YES	NO	One	Few	Many
a.	Slapped you or thrown something at you that could hurt you?	1	2	1	2	1	2	3
b.	Pushed you or shoved you or pulled your hair?	1	2	1	2	1	2	3
c.	Hit you with his fist or with something else that could hurt you?	1	2	1	2	1	2	3
d.	Kicked you, dragged you or beaten you up?	1	2	1	2	1	2	3
e.	Choked or burnt you on purpose?	1	2	1	2	1	2	3
f.	Threatened to use or actually used a gun, knife or other weapon against you?	1	2	1	2	1	2	3
g.	Has hit you or beaten you with (hard) objects such as belts, hairbrush or canes?	1	2	1	2	1	2	3

**514**	SKIP IF 512 ALL NO<br>Was the person who did these things to you your current partner, or a previous partner?	Current<br>Previous/Past<br>Both	1<br>2<br>3

515-516		**515** **(If YES continue with 515x. If NO go to next 515 sub question, if ALL NO skip to 518)**		515x. Has this happened in the past 12 months? (If YES ask 516. If NO ask 518)		**516** <u>In the past 12 months</u> would you say that this has happened once, a few times or many times?		
		YES	NO	YES	NO	One	Few	Many
a.	Has <u>your current husband/partner or any other partner ever</u> forced you to have sexual intercourse by threatening you, holding you down or hurting you in some way?	1	2	1	2	1	2	3

b.	Have you ever had sexual intercourse when you did not want to because you were afraid that your partner would hurt you or someone you cared about if you refused?	1	2	1	2	1	2	3
c.	Have you ever had sexual intercourse when you did not want to because you were afraid that your partner would leave you or take another girlfriend if you refused?	1	2	1	2	1	2	3
d.	Has your husband/partner ever insisted on having anal sex although he knew you did not want to?	1	2	1	2	1	2	3
e.	Has your husband/partner ever insisted on having oral sex although he knew you did not want to?	1	2	1	2	1	2	3

**517**	SKIP IF 515 ALL NO<br>Was the person who did these things to you your current partner, or a previous partner?<br><br>IF RESPONSES TO QUESTIONS 509, 512 AND 515 ARE ALL NO, SKIP QUESTIONS 518 AND 519	Current<br>Previous/Past<br>Both	1<br>2<br>3

**518**	In the past 12 months, during these incidents of violence did you ever fight back physically to defend yourself?	Yes<br>No<br>No partner in the past 12 months	1<br>2<br>3	 520<br>533
**519**	IF YES: READ OUT ANSWERS	Once<br>Few times<br>Many times<br>Don't know<br>Refuse to answer	1<br>2<br>3<br>96<br>99	
**520**	In the past 12 months did you ever hit or physically mistreated your husband/partner when he was not hitting or physically mistreating you?	Yes<br>No	1<br>2	 522
**521**	IF YES: READ OUT ANSWERS	Once<br>Few times<br>Many times<br>Don't know<br>Refuse to answer	1<br>2<br>3<br>96<br>99	

	QUESTIONS	CODING CATEGORIES		
**522**	Thinking back over the past 12 months, would you say that you have been very afraid of your partner?<br><br>Would you say, never, a few times, many times or most of the time?	Never<br>a few times<br>Many times<br>Most /all of the time<br>Refuse to answer	1<br>2<br>3<br>4<br>99	
**523**	In the past 12 months, how often have children living in your household seen or heard you being beaten by your partner?<br>Would you say, never, happened few times,many times or most /all of the time….<br><br>IF THE RESPONSE TO QUESTION 120 IS NO , SKIP THIS QUESTION	Never<br>Few times<br>Many times<br>Most /all of the time<br>Refuse to answer	1<br>2<br>3<br>4<br>99	

If YES to any sub-question in 509, 512 or 515, continue. If ALL NO, SKIP to SECTION 6.

From your answers I can see that you have had some difficulties with your current partner or past partners. Now I would like to ask learn more about what you have done to deal with these difficult situations in the last 12 months.

**524**	In the last 12 months, who have you told about your partner's behaviour?	YES	NO
a.	Friend	1	2
b.	Parents	1	2
c.	Brother or sister	1	2
d.	Uncle or aunt	1	2
e.	Husband/partner's family	1	2
f.	Children	1	2
g.	Neighbours	1	2
h.	Police	1	2
i.	Doctor/health worker	1	2
j.	Religious leaders	1	2
k.	Counsellor	1	2
l.	NGO/women's organization	1	2
m.	Local leader	1	2
n.	Member of loan group	1	2
o.	BRAC staff	1	2
p.	OTHER (specify):________________________	1	2
q.	No one	1	2
r.	Traditional healer	1	2
**525**	In the last 12 months, did anyone ever try to help you?	YES	NO
a.	Friend	1	2
b.	Parents	1	2
c.	Brother or sister	1	2
d.	Uncle or aunt	1	2
e.	Husband/partner's family	1	2
f.	Children	1	2
g.	Neighbours	1	2
h.	Police	1	2
i.	Doctor/health worker	1	2
j.	Religious leaders	1	2
k.	Counselor	1	2
l.	NGO/women's organization	1	2
m.	Local leader	1	2
n.	Member of loan group	1	2
o.	BRAC staff	1	2
p.	OTHER (specify):________________________	1	2
q.	No one	1	2
r	Traditional healer	1	2
**526**	In the last 12 months, did you ever go to any of the following for help?<br>READ EACH ONE	YES	NO
a.	Police	1	2
b.	Hospital or health centre	1	2
c.	Social services	1	2
d.	Legal advice centre	1	2
e.	Court	1	2
f.	Shelter	1	2
g.	Street leader	1	2

h.	Women's organization: Name ,specify________________	1	2
i.	Priest/Religious leader	1	2
j.	Anywhere else? Where? specify___________________	1	2
k.	Traditional healer	1	2

**527**	Have you left in the past 12 months, even if only for a night, because of his behaviour?	Yes<br>No	1<br>2	 532a
**528**	If so, how many times?	[ ][ ]		
**529**	How long did you stay away the **last time you left**?	One day<br>Several days<br>Months<br>Left Partner	1<br>2<br>3<br>4	
**530**	Where did you go?	Family<br>Friends/Neighbour's<br>Hotel<br>Other specify....................	1<br>2<br>3<br>4	
**531**	Did you return?	Yes<br>No	1<br>2	 532a
**532**	[IF RETURNED] Why did you return?	I did not want to leave Children<br>Sanctity of Marriage<br>Family said to return<br>Did not know how to support myself<br>Forgave him<br>He threatened me<br>Other specify__________________	1<br>2<br>3<br>4<br><br>5<br>6<br>7	

532a.	Before the age of 18 has anyone ever forced you to have sex or perform a sexual act when you did not want to?	Yes ___1<br>No ___2<br>No answer ___3	532c
532b.	Who did this to you?	Father<br>Stepfather<br>Other male family member<br>Teacher<br>Police<br>Male friend of the family<br>Boyfriend<br>Stranger<br>Someone at work<br>Priest<br>Husband/sex partner<br>Other<br>Mention:................	1<br>2<br>3<br>4<br>5<br>6<br>7<br>8<br>9<br>10<br>11<br>12

		If Yes, ask 532x				532x If yes, did this happen to you in the last year		
		Yes	No	NA	Refused/No answer	Yes - Once	Yes – Few times	Yes – Often (many times)
532c.	Has anyone ever made sexually suggestive comments or jokes that made you feel offended?							
532d.	Have you ever received personal electronic messages with sexual content (e.g. remarks, invitations, pictures) that were hurtful to you or made you feel uncomfortable? For example, via Facebook, cellphone, e-mail, excluding spam							
532e.	Have you ever been asked to perform sexual acts against your will in order to get a job or keep your job, or to get promoted?							
532f.	Have you ever been asked to perform sexual acts against your will in order to pass an exam or get good grades at school? (The acts do not need to have happened).							
532g.	Have you ever been groped, sexually touched or had someone rubbing against you in the bus or another public space?							

533	**(If she answered SHE HAS NO CHILD on question 120, go to section 6)** Adults use certain ways to teach children the right behavior or to address a behavior problem. I will read various methods that are used. Please tell me if you or your partner has used this method with any of the children in your household in the past 12 months. Indicate one or more of You and your Partner	Participant		**534** Partner	
a	Took away privileges, forbade something they liked or did not allow him/her to leave the house?	Yes<br>No	1<br>2	Yes<br>No	1<br>2
b	Explained why your child's behavior was wrong?	Yes<br>No	1<br>2	Yes<br>No	1<br>2
c	Shook him/her	Yes<br>No	1<br>2	Yes<br>No	1<br>2
d	Shouted, yelled at or screamed at him/her	Yes<br>No	1<br>2	Yes<br>No	1<br>2
e	Gave him/her something else to do	Yes<br>No	1<br>2	Yes<br>No	1<br>2
f	Spanked, hit or slapped him/her on the bottom with bare hand	Yes<br>No	1<br>2	Yes<br>No	1<br>2
g	Hit him/her on the bottom or elsewhere on the body with something like a belt, hairbrush, or other hard object	Yes<br>No	1<br>2	Yes<br>No	1<br>2
h	Called him/her dumb, lazy or another name like that	Yes<br>No	1<br>2	Yes<br>No	1<br>2
i	Hit or slapped him/her on the face, head or ears	Yes<br>No	1<br>2	Yes<br>No	1<br>2
j	Hit or slapped him/her on the hand, arm or leg	Yes<br>No	1<br>2	Yes<br>No	1<br>2
k	Beat him/her up, that is hit him/her over and over as hard as one could	Yes<br>No	1<br>2	Yes<br>No	1<br>2
l	Do you believe that in order to bring up, raise, or educate a child properly, the child needs to be physically punished?	Yes<br>No	1<br>2	Yes<br>No	1<br>2

SECTION 6: ABOUT YOUR PARTNER'S CHILDHOOD

Check question 106 – if NO,

QUESTIONS	CODING CATEGORIES

PLEASE LOOK AT QUESTION 106. IF RESPONSE IS NO – ASK QUESTIONS 600 TO 604 ABOUT HER CURRENT PARTNER; IF YES, SKIP TO SECTION 7.

I would like to ask few questions about things that could have happened to your current husband/partner when he was a child.

600.	As far as you know, when your current partner was a child, did he live with a household member who was an alcoholic in your household?	Yes<br>No<br>Don't know<br>No partner	1<br>2<br>96<br>99
601.	As far as you know, when your current partner was a child, did he live with a household member who was sent to jail	Yes<br>No<br>Don't know<br>No current partner	1<br>2<br>96<br>99
602.	As far as you know, when your current partner was a child, did his mother, father or guardian die?	Yes<br>No<br>Don't know<br>No partner	1<br>2<br>96<br>99
603.	As far as you know, when your current partner was a child, was he beaten himself regularly by someone in his family?	Yes<br>No<br>Don't know<br>No partner	1<br>2<br>96<br>99
604.	As far as you know, when your current partner was a child, was he beaten so hard that left marks or injured?	Yes<br>No<br>Don't know<br>No partner	1<br>2<br>96<br>99

SECTION 7: ABOUT YOUR COMMUNITY

I would now like to learn a bit more about the groups and organizations that you are part of in your community.

	QUESTIONS	CODING CATEGORIES				
700-701	Please tell me if you are part of the following community groups?	700.		701. If **YES for question 700**, please also tell me if you are simply attending, if you are actively contributing or even leading some of the group activities.		
		Yes	No	Member /attends	Active	Leader
a.	Religious group	1	2	1	2	3
b.	Ethnic groups/Cultural groups	1	2	1	2	3
c.	Economic support groups (non-finance)	1	2	1	2	3
d.	*Mtaa* based groups	1	2	1	2	3
e.	Celebration/Burial help groups (vikundi vya sherehe/ na kuzikana)	1	2	1	2	3
f.	Women's group (Non finance)	1	2	1	2	3
g.	Credit/finance group	1	2	1	2	3
h.	Economic finance group (finance)	1	2	1	2	3
i.	Others (specify) ____________________	1	2	1	2	3

**702**	Is your partner involved in a community group with you or in another one?	Yes - same<br>Yes – another one<br>No<br>Don't know<br>No partner	1<br>2<br>3<br>4<br>5

	QUESTIONS	CODING CATEGORIES	
	When I read the following statements can you please say whether you personally AGREE OR DISAGREE,<br><br>PROBE WHERE NEEDED.<br><br>READ SENTENCE AND ASK IF AGREE , OR STRONGLY AGREE.<br><br>IF DISAGREE, THEN, ASK IF DISAGREE or STRONGLY DISAGREE.		
**703**	Neighbors in this community tend to know each other well	Strongly agree<br>Agree<br>Disagree<br>Strongly disagree	1<br>2<br>3<br>4
**704**	In this community, people, generally trust each other in matters of lending and borrowing	Strongly agree<br>Agree<br>Disagree<br>Strongly disagree	1<br>2<br>3<br>4

**705**	In this area, it is safe to walk around at night.	Strongly agree<br>Agree<br>Disagree<br>Strongly disagree	1<br>2<br>3<br>4
**706**	In this area, people lend a hand when somebody dies.	Strongly agree<br>Agree<br>Disagree<br>Strongly disagree	1<br>2<br>3<br>4
**707**	In the past year, have you participated in a meeting, march, rally or gathering aiming to raise awareness and mobilize people around an issue that is important in your community? For example, HIV, rights for albinos or women, etc.	Yes<br>No	1<br>2
**708**	Have you spoken out in public in the past 12 months?	Yes<br>No	1<br>2
**709**	Have you intervened in cases of domestic violence in the past 12 months?	Yes<br>No	1<br>2
	To conclude, I would like to ask you a few questions about the gender training provided by MAISHA		
**710**	Have you attended any of the MAISHA gender training sessions?	Yes<br>No	1<br>2
**711**	How many sessions of MAISHA training did you attend?	Number of sessions:	[ ][ ]
**712**	Which sessions did you find the most helpful for you personally and for your relationship?<br>(Indicate ALL that apply)	Taja:<br>..........................<br>..........................<br>..........................<br>..........................<br>..........................	 1<br>2<br>3<br>4<br>5

WHEN YOU ARE DONE

Interview details – END

Date of interview:

Time interview ended:

Name of interviewer:

Are you the same interviewer as at the beginning? No **Yes**

Comments:

Interview closure

Finish one – Respondent <u>has disclosed</u> problems/violence/sexual harassment

I would like to thank you very much for helping us. I appreciate the time that you have taken. I realize that these questions may have been difficult for you to answer, but it is only by hearing from women themselves that we can really understand about their situation, and how to help them.

From what you have told us, I can tell that you have had some very difficult times in your life. No one has the right to threaten someone else in that way. However, from what you have told me I can see that you are strong, and have survived through some difficult circumstances.

Here is a list of organizations that provide support, legal advice and counseling services to women in Mwanza. Please do contact them if you would like to talk over your situations with anyone. Their services are free, and they will keep anything that you say private. You can go whenever you feel ready to, either soon or later on. We would be happy to help you contact them, if this would be helpful to you.

Finish two – Respondent <u>has not disclosed</u> problems/violence/sexual harassment

I would like to thank you very much for helping us. I appreciate the time that you have taken. I realize that these questions may have been difficult to answer, but it is only by hearing from women themselves that we can really understand about women's health and experiences in life.

In case you ever hear of another woman who needs help, here is a list of organizations that provide support, legal advice and counseling services to women in Mwanza. Please do contact them if you or any of your friends or relatives needs help. Their services are free, and they will keep anything that anyone says to them private.

Finish – Informing them that this is the end of study

This interview completes your participation in the MAISHA longitudinal study. You will not be interviewed again although we might contact you in case of any relevant information related to this study. We may also contact you in the future if we have another study but you will need to provide a fresh consent for that. We thank you for your participation and wish you all the best.
